# Antimicrobial Activity of Poly(methyl methacrylate) Doped with CuO and ZnO Nanoparticles

**DOI:** 10.1021/acsomega.4c10170

**Published:** 2025-03-28

**Authors:** Nives Matijaković Mlinarić, Anamarija Zore, Valentina Veselinovic, Nataša Trtić, Olivera Dolić, Roman Štukelj, Anže Abram, Aleksander Učakar, Tijana Adamović, Rajko Vidrih, Franc Rojko, Kaja Kasemets, Anne Kahru, Klemen Bohinc

**Affiliations:** † Faculty of Health Sciences, University of Ljubljana, Zdravstvena pot, 1000 Ljubljana, Slovenia; ‡ Faculty of Medicine, University of Banja Luka, 78000 Banja Luka, Bosnia and Herzegovina; § Jožef Stefan Institute, Jamova cesta 39, 1000 Ljubljana, Slovenia; ∥ Biotechnical Faculty, University of Ljubljana, Jamnikarjeva ulica 101, 1000 Ljubljana, Slovenia; ⊥ Laboratory of Environmental Toxicology, National Institute of Chemical Physics and Biophysics, Akadeemia tee 23, Tallinn 12628, Estonia

## Abstract

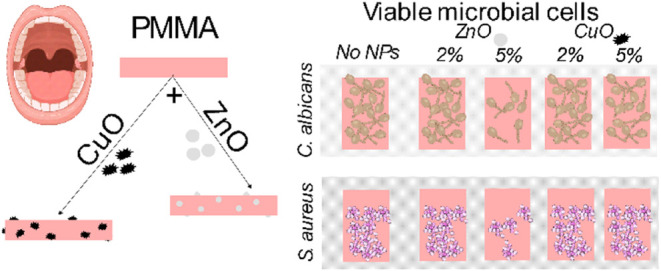

Oral health represents a significant factor in general health and life quality. A significant number of people are affected by tooth loss during their lifetimes, especially in the older population. Poly(methyl methacrylate) (PMMA) resins are the preferred option for replacing missing teeth due to the material stability, easy handling, low toxicity, and most importantly biocompatibility with human tissue. Even though PMMA is the preferable material for denture preparation, it is susceptible to microbial colonization, which can induce the development of oral infections. This study aimed to increase the antimicrobial effect of PMMA and compare the antimicrobial properties of PMMA incorporated with different amounts (2 and 5 wt %) of zinc oxide (ZnO; primary size 62.4 nm ± 16.7 nm) and copper oxide (CuO; primary size 434.0 nm ± 118.5 nm) nanoparticles to determine their antimicrobial effects on Gram-positive bacteria *Staphylococcus aureus* and yeast *Candida albicans*—pathogenic microbes often found on dentures. To understand the adhesion of microorganisms to PMMA-modified surfaces, the following surface properties were measured: roughness, contact angle, and ζ potential. In addition, CIE (the International Commission on Illumination) color parameters of the materials were determined. The bacterial adhesion was measured by viable plate counts and scanning electron microscopy. Our study showed that 5 wt % ZnO added to PMMA yields a promising denture material that is esthetically acceptable and shows antimicrobial properties toward both, *Staphylococcus aureus* and *Candida albicans*.

## 1 Introduction

Oral diseases affect people throughout the world and pose a major health burden affecting nearly 3.5 billion people.^1^ Diseases like periodontitis, dental caries, tooth trauma, and other causes can lead to tooth loss. Globally around 7% of people aged 20 years or older suffer complete teeth loss, while the prevalence is much higher (23%) in people older than 60 years.^2,3^ According to data from the World Health Organization, the share of the world’s population over 60 will almost double from 12 to 22% in the period between 2015 and 2050.^4^ In line with the increase in the elderly population, there is also an increase in the number of partially or completely toothless people. The dental profession is faced with the challenges of how best to treat such patients and how to adapt therapeutic procedures to meet the basic standards of therapeutic success such as the restoration of function, aesthetics, and improvement of quality of life.^5^ Partial or total dentures, as a prevalent method of prosthetic rehabilitation, represent gingival-supported restoration, which achieves close contact with the soft tissues of the oral cavity with a large surface area. Poly(methyl methacrylate) has been the material of choice in the production of mobile prostheses for many years.^6,7^

PMMA resins are widely used in dental medicine, mostly because of their outstanding mechanical properties, simple denture production, cost-effectiveness, low density and low weight, satisfactory aesthetics and color matching, stability in the oral cavity, and most importantly, biocompatibility with human tissue.^8,9^ Although PMMA resins have excellent properties when used as dental materials, they are still susceptible to bacterial and fungal colonization.^10^ Especially since the dental cavity is ideal for microbial development and growth due to suitable conditions such as temperature, moisture, and available food sources.^11^

Prosthetic stomatitis is a chronic inflammatory process of denture underlying soft tissues, and it can develop in as many as 67% of denture wearers.^12^ The most common denture stomatitis causative agents are fungi from Candida species and mechanical trauma caused by inadequate dentures. Although Candida is part of the physiological flora of the oral cavity, under certain conditions, it can become pathogenic and even lead to systemic reactions and death. Resistance to conventional drugs of *Candida albicans* (*C. albicans*) is growing. The World Health Organization (WHO) has placed it in the critical priority group as part of the WHO fungal priority pathogens list (WHO FPPL), as a high-risk fungal pathogen, whose invasive infections can cause death in 20–50% of cases regardless of the applied therapy.^13^ According to WHO, on the list of bacterial priority pathogens (BPPL), for which it is necessary to urgently develop new effective drugs, *Staphylococcus aureus* (*S. aureus*) is marked as a high-priority pathogen.^13^ The Global Burden of Disease study reported that approximately 50% of the fatal burden associated with antimicrobial resistance is linked with *S. aureus* and *Escherichia coli* (*E. coli*).^14^*S. aureus* can be both a commensal and a pathogenic microorganism that causes serious infections with a potentially fatal outcome. Although the nasal cavity is considered the primary host of *S. aureus*, the oral cavity is the site of colonization with approximately the same prevalence (13.9 and 12.0%) and similar rates of antibiotic resistance (83.3–81.5%).^15^

Modern studies deal with different approaches to reducing the risk of infection in the wearer of total dentures, among which the researchers’ special attention is drawn to the modification of conventional acrylates for the manufacture of the denture base by the addition of nanoparticles (NPs) such as silver nanoparticles (AgNPs), titanium dioxide (TiO_2_NPs), zinc oxide (ZnO NPs), zirconium dioxide (ZrO_2_NPs), silicon dioxide (SiO_2_NPs), copper oxide (CuONPs), and similar.^16^ Among other characteristics, satisfactory aesthetics is required for dentistry as a restorative material. Restorative material should match as closely as possible the color of the human target tissue, i.e., gingiva. The addition of different nanoparticles to poly(methyl methacrylate) (PMMA) might significantly impact color; TiO_2_ nanoparticles increased lightness and decreased both red and yellow color components.^17^ On the other hand, the addition of Au nanoparticles only slightly changed the color of PMMA.^18,19^ One of the focuses of the scientific community’s activities is the use of metal nanoparticles in numerous biomedical applications. The advantage of the antimicrobial effect of nanoparticles compared to conventional drugs is their nonspecific mechanisms of antibacterial activity. The fact that nanoparticles do not bind to a specific receptor in a bacterial cell makes it difficult for microorganisms to develop resistance and provides nanoparticles with a wider spectrum of antimicrobial activity. As a result, the vast majority of efficacy studies of metal-based nanoparticles to date have shown promising results in both Gram-positive and Gram-negative bacteria.^20^

The focus of our research was directed to zinc oxide (ZnO) and copper oxide (CuO) nanoparticles which in general exhibit good antimicrobial and antifungal activity.^21−24^ Tasnim et al. demonstrated that the enhanced antimicrobial activity and physicochemical properties benefit from metal doping. In the context of selecting Zn and Cu, these metals are particularly advantageous because both ZnO and CuO are well-documented for their strong antimicrobial effects, including ROS generation, which is crucial for disrupting microbial cells; Zn and Cu exhibit lower toxicity at optimized concentrations compared to some other metals, making them suitable for biomedical applications like PMMA-based materials; Zn and Cu doping improves surface properties, such as charge density and interaction with microbial cells, leading to enhanced antimicrobial efficiency.^25^ ZnO nanoparticles are safe, biocompatible, have a strong antibacterial effect on a broad range of Gram-negative and Gram-positive bacteria and fungi as well increase the flexural strength, decrease the shear bond strength, and compressive strength when used in composites and resins.^26−28^ These properties make them particularly advantageous for clinical applications such as dentures, where safety and long-term material stability are critical. The PMMA doped with ZnO has shown promising antifungal effects against *C. albicans*(29,30) and acceptable aesthetics regarding the color modification of the doped PMMA with 2 to 5 wt % weight content of ZnO nanoparticles.^31^ Analogously, PMMA resins doped with CuO/TiO_2_ nanoparticles showed effective antimicrobial activity against *Streptococcus salivarius*, *Streptococcus sanguis*, and *Candida dubliniensis*.^32^ Moreover, the addition of copper nanoparticles in concentrations of 25 ppm in denture resins showed high flexural strength and an inhibitory effect against yeast *C. albicans* biofilm formation.^33^

Copper is a semiconductor material that, due to its heat resistance, stability, economy, and uncomplicated synthesis, is considered a very good candidate for the synthesis of metal-based nanoparticles. Its antimicrobial properties have been proven in numerous studies.^34−36^ Sathya et al. studied PMMA doped with CuO and ZnO and showed that CuO nanoparticles were more effective against Gram-negative bacteria *Escherichia coli*, while the ZnO was more effective against Gram-positive bacteria *S. aureus*.^37^ However, due to the prevalence of oral candidiasis and the fact that 80% of the general population are asymptomatic carriers,^11^ it is important to investigate and compare the inhibitory properties of CuO and ZnO also toward yeast *C. albicans*.

There is limited research comparing ZnO and CuO effects when incorporated into PMMA resin against both *S. aureus* and *C. albicans*. While the antibacterial effects of ZnO and CuO have been explored previously,^37^ research on their comparative activity against yeast, specifically *C. albicans*, when incorporated into PMMA, remains scarce. By including *C. albicans* in this study, new insights into the dual antimicrobial activity of these nanoparticles could highlight their potential to address both bacterial and fungal colonization on denture surfaces. Thus, this study aimed to compare the antimicrobial activity of PMMA doped with CuO and ZnO (2 and 5 wt %, respectively) on bacteria *S. aureus* and yeast *C. albicans* to elucidate the inhibitory effect on microbial cells on the PMMA surfaces.

## 2 Materials and Methods

### 2.1 Nanoparticle Preparation and Characterization

CuO and ZnO nanoparticles (CuO NPs, ZnO NPs) were prepared as previously described.^38,39^ Shortly, analytical grade chemicals: CuSO_4_·5H_2_O (0.1 mol dm^–3^), Zn(CH_3_CO_2_)_2_·2H_2_O (0.1 mol dm^–3^), and NaOH (1.0 mol dm^–3^) (Sigma-Aldrich) without further purification were used. CuO NPs were prepared by mixing NaOH solution (*V* = 0.5 mL) with CuSO_4_ solution (*V* = 2 mL) at 60 °C. The suspension was magnetically stirred (300 rpm) until the color of the suspension changed from blue to black. The Zn(CH_3_CO_2_)_2_ solution was prepared by dissolving an appropriate ionic salt in a 5:1 solution of water and ethanol. ZnO NPs were prepared by adding 50 mL of NaOH to 150 mL of Zn(CH_3_COO)_2_ solution. The suspension was heated to 70 °C and mixed magnetically for 15 min and left overnight. The obtained NPs were separated from the suspension by centrifugation at 6000 rpm for 10 min and washed with deionized water in several cycles. The collected sample was dried at 100 °C for 5 h and annealed in an oven at 400 °C for 2 h.

The composition of the nanoparticles was confirmed by Fourier-transform infrared (FTIR) spectra and X-ray diffraction on the polycrystalline sample (XRD). The FTIR spectrum was measured on PerkinElmer FTIR C89391 (PerkinElmer, MA) in the range of 300–1800 cm^–1^. The XRD diffractions were measured on an Aeris Panalytical diffractometer (Malvern Panalytical, Malvern, United Kingdom) with Ni-filtered copper radiation, in Bragg–Brentano geometry in the 2θ range 30–80° with step size 0.01°, 1 s per step. The obtained diffraction pattern was analyzed by the PANalytical High Score Plus software. The nanoparticle morphology was determined by scanning electron microscopy (SEM) on a JEOL 7600 (JEOL Ltd., Akishima, Tokyo, Japan) with a thin layer of gold. The mean CuO and ZnO particle size was determined by dynamic light scattering (DLS), in water at room temperature, using the Zetasizer Ultra (Malvern Panalytical, Malvern, United Kingdom). The DLS analysis was performed with 30 mg mL^–1^ of the sample (in quintuplicates) with a total volume of 1.0 mL in a disposable DTS0012 plastic cell. To determine the NP hydrodynamic diameters, the Einstein-Stokes equation was used.

### 2.2 Poly(methyl methacrylate) Composites Preparation

PMMA samples were made in insulated gypsum molds, which served as a compressor for compressing the PMMA mass. PMMA ProBased Cold polymer and ProBased Cold (Ivoclar Vivadent Inc.) monomer were mixed in a ratio of 10 mL of monomer to 15 g of polymer powder, stirred well, and allowed to sit for a few minutes to reach the prepolymerization phase. The mixture was kneaded and placed into the gypsum molds when it reached a dough-like consistency. The mixture was compressed using a hydraulic press or a specific device (MOFI: model fixture). The specimens were then placed into a polymerization bath with lukewarm water (40 °C) under a 20 bar pressure. The polymerization process lasted 15 min. The polymerized specimens were removed from the gypsum molds. For the samples with CuO and ZnO NPs during the compression step of the PMMA, a 1 mm thick layer of insulating material (polyvinyl film) was applied to create a space. The PMMA modified with NPs, ZnO, and CuO were mixed with the polymer in the powder form to obtain 2 and 5 wt % of NPs in the PMMA mixture. After the application of the nanoparticle-containing PMMA layer, the gypsum mold was compressed and polymerization was conducted. The PMMA mixture was polymerized and prepared in (1 × 1 × 0.3) cm pieces. Before the adhesion test, the samples were washed with 70% ethanol and sterilized under UV light for 30 min. After the surface properties and antimicrobial activity analysis, the samples were washed three times with 5 mL of DI water and then soaked for 15 min in 5 mL of 70% ethanol (in a 6-well plate) and sterilized under UV light for 30 min. Samples were used in duplicates or triplicates.

### 2.3 Contact Angle Measurements

The Attension Theta Tensiometer (Biolin Scientific AB, Gothenburg, Sweden) was used to determine the static water contact angle on the specimens’ surfaces by employing the sessile drop technique. A water droplet (5 μL) was seeded on the surface from the tip of the needle (diameter = 0.4 mm), and the static water contact angle was determined on the three-phase boundary.

### 2.4 ζ Potential

The ζ potential of the surfaces was measured on a SurPASS electrokinetic analyzer (Anton Paar GmbH, Graz, Austria) at pH 6.5 and room temperature in a 1 mmol dm^–3^ potassium chloride.^40^

### 2.5 Color Determination

The color of the untreated and doped PMMA was measured with Minolta spectrophotometer CM-5. The data were analyzed according to the *Commission Internationale de l’Eclairage* (CIE), and measurements were expressed in L*, a*, b* color parameters. Each sample was measured in triplicate.

### 2.6 Antimicrobial Studies

The bacterial culture was prepared by transferring a colony of *Staphylococcus aureus* ATCC 25923 (*S. aureus*) into 5 mL of brain heart infusion growth medium (Biolife Italiana, Milano, Italy). The fungal culture was prepared by transferring one colony of *Candida albicans* (*C. albicans*), a clinical strain obtained from the Institute of Microbiology and Immunology, University of Ljubljana, into 5 mL of Sabouraud nutrient broth (Biolife Italiana, Milano, Italy). The prepared suspension was incubated overnight at 37 °C without shaking. The optical density of the overnight cultures was measured at OD_600_ following the well-established method of correlation of optical density (OD) measurements and colony-forming unit (CFU) counts,^41,42^ to achieve a final concentration of 10^5^ CFU/mL. The optical density was measured in 96 well plates in triplicate on a spectrophotometer (Tecan, Mannedorf/Zürich, Switzerland) at a wavelength of 600 nm. Prior to experiments, it was determined that for *S. aureus* OD600 value of 0.05 is approximately equal to 1 × 10^6^ CFU/mL, while for *C. albicans* OD600 value of 0.28 is approximately equal to 1 × 10^6^ CFU/mL. To prepare the 10^5^ CFU/mL, the *S. aureus* suspension with 0.05 OD600 and *C. albicans* of 0.28 OD600 were ten times diluted in the respective growth medium. The 1 cm × 1 cm PMMA samples were sterilized under UV light and placed in a 24-well plate. The experiments were conducted in triplicate, n = 3. The PMMA without CuO or ZnO was used as a negative control. The positive controls were included during the experiments: for *S. aureus*, a diluted suspension of overnight culture in BHI medium with erythromycin (1 μg/mL), and for *C. albicans*, a diluted suspension in Sabouraud medium with Nystatin (32 units/mL) were used. The positive controls demonstrated complete growth inhibition of *S. aureus* and *C. albicans*, validating the experimental protocol by producing the expected result, namely, no viable fungal or bacterial cells were present after 24 h of incubation. Antimicrobial testing was conducted by adding 100 μL of the diluted bacterial or yeast suspension onto the surface of the samples in triplicate and incubating for 24 h at 37 °C. After incubation, the samples were placed into 0.9% NaCl solution (2.5 mL) and sonicated in a sonication water bath for 5 min, followed by vigorous vortexing for 15 s at the highest speed for the detachment of cells from the PMMA surface. After a series of dilutions, 10 μL of the *S. aureus* and *C. albicans* suspensions were loaded on plate count agar plates and incubated for 18 h at 37 °C.

The antimicrobial efficiency was expressed as the percentage reduction in the viable microbial cells, eq 1
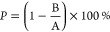
1from the number of viable microbial cells before the addition of NPs (A) and the number of viable microbial cells after the NPs application (B).

The extent of adherence of cells to the PMMA surface was determined with scanning electron microscopy (SEM) following the modified procedure from ref (43). After the incubation, the nonadhered cells were washed off with 1 mM phosphate-buffered saline (PBS) solution. The adhered cells were fixed on the PMMA surface with hot air at 60 °C for 10 min. Then the samples were washed with distilled water to remove PBS crystals, and the drying was repeated for 10 min at 60 °C. Before the SEM analysis on the surface of the samples, a thin gold layer (7 nm) was applied to the GATAN Model 682 PECS system (Precision Ion Etching and Coating System, GATAN Inc., Pleasanton, CA). The adhered cells were visualized on SEM Jeol JSM-7600F (Jeol, Tokyo, Japan).

### 2.7 Statistical Analysis

All experiments were conducted in triplicate. The results were statistically analyzed by using the GraphPad Prism 8 (La Jolla, CA). A one-way analysis of variance (ANOVA) was applied to evaluate the statistical significance of the conducted experiments by using Tukey’s multiple comparison test. Statistical significance was considered for the p-value below 0.05, i.e., 95% of the confidence interval.

## 3 Results

**Figure 1:**
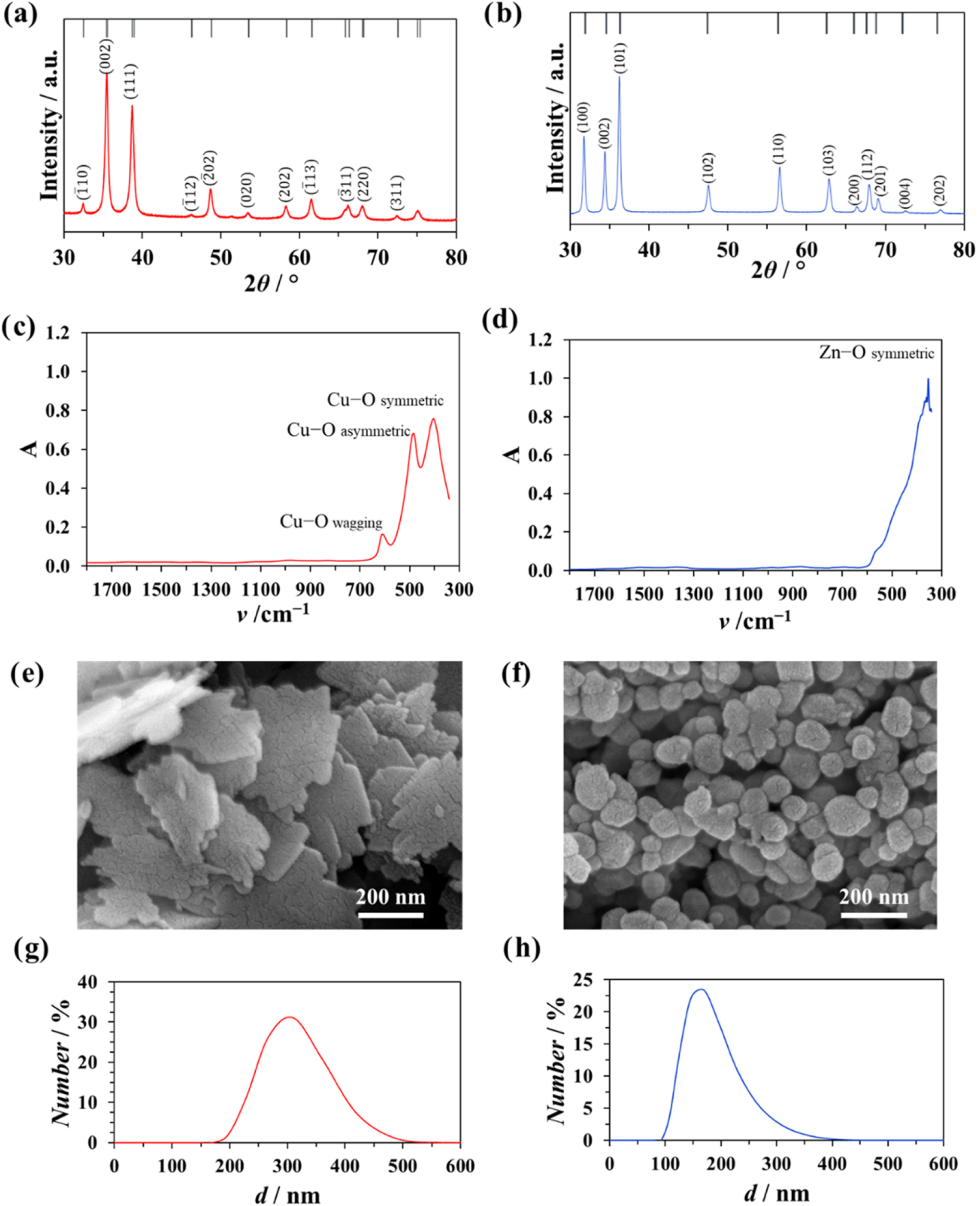
Characterization of CuO and ZnO nanoparticles annealed at 400 °C. X-ray diffractogram of the prepared CuO (a) and ZnO (b) NPs and main reflections of CuO and ZnO shown for comparison; FTIR spectra with assigned bond vibrations where A and ν indicate absorbance and wavelength, respectively, for (c) CuO and (d) ZnO NPs and the morphology of (e) CuO and (f) ZnO NPs. The particle size distribution *d* is shown for (g) CuO NPs and (h) ZnO NPs.

CuO and ZnO NPs were prepared as published before with some modifications,^38,39^ and the obtained NPs were analyzed by XRD analysis to confirm the composition. In the diffraction pattern of CuO NPs (Figure 1a), peaks were assigned to pure CuO, as confirmed by JCPDS card number 80-1917. Furthermore, in the diffractogram of ZnO NPs (Figure 1b), peaks corresponding to ZnO were confirmed based on the JCPDS card number 36-1451.

The FTIR analysis of CuO NPs (Figure 1c) revealed peaks at 403, 485, and 608 cm^–1^ which can be assigned to symmetric stretching, antisymmetric stretching, and wagging of the Cu–O bond.^44^ In the spectrum of ZnO NPs (Figure 1d), the most significant peak in the region below 400 cm^–1^ can be assigned to symmetric stretching of the Zn–O bond.^45^

Furthermore, scanning electron microscopy was used to determine the morphology and size of the NPs. The CuO NPs (Figure 1e) were prepared as thin sheets with rugged edges,^38^ while ZnO NPs (Figure 1f) were prepared as irregular spheres. The primary size of the CuO and ZnO nanoparticles was determined from SEM micrographs (Figure 1e,1f). The size of ZnO NPs was (62.4 ± 16.7) nm, whereas the size of CuO particles was (434.0 ± 118.5) nm. CuO particles exhibited a hydrodynamic diameter of (325 ± 104) nm (Figure 1g), which was in accordance with the particle size determined from the SEM. The ZnO NPs exhibited a smaller and narrower hydrodynamic diameter of approximately (140 ± 40) nm (Figure 1h), the much smaller nanoparticles were visually aggregated in the water suspension, and the exact size of the NPs was determined by analyzing the SEM images as shown above. The prepared CuO and ZnO were used in the preparation of the doped PMMA resins.

**Figure 2:**
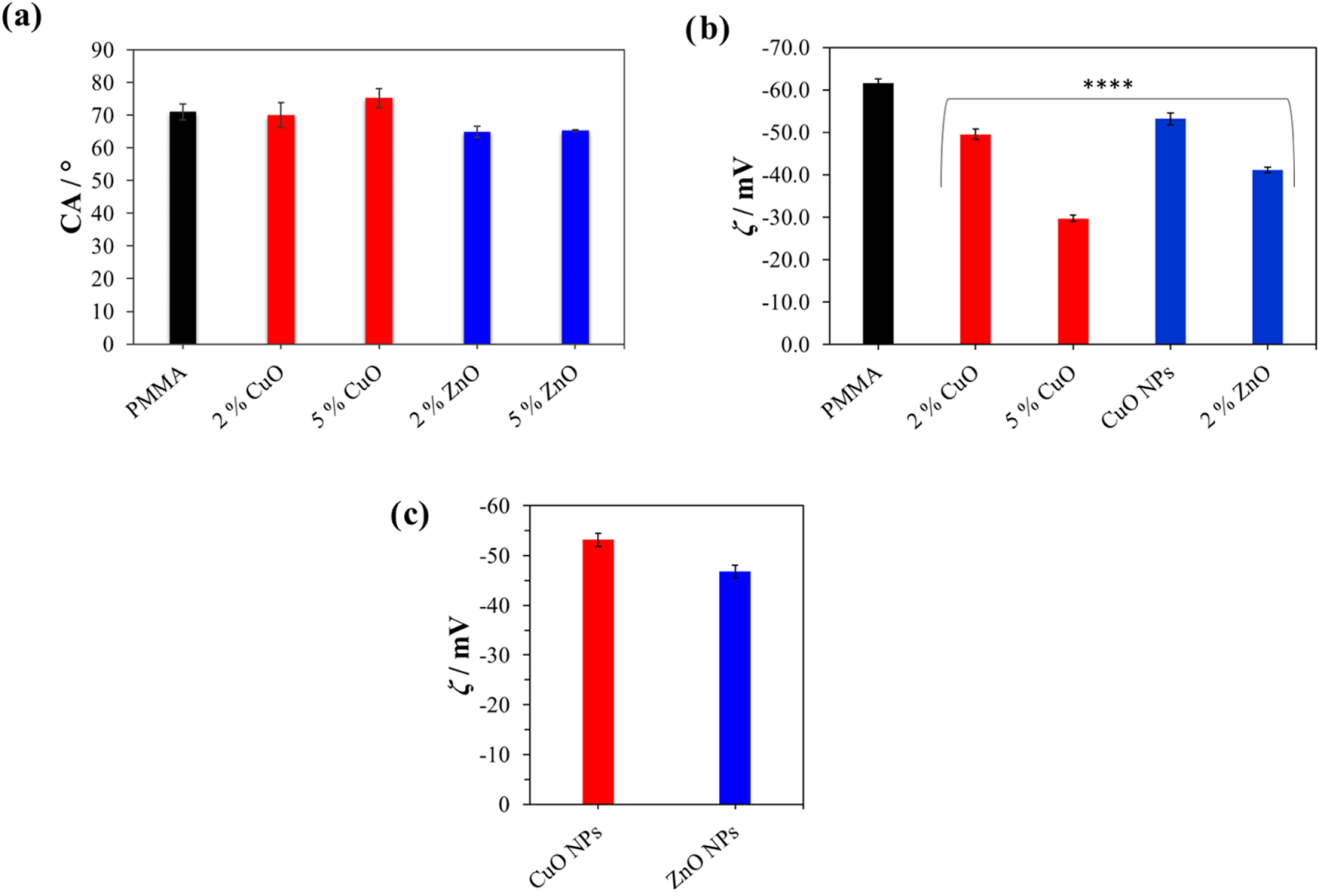
Water contact angles (CA) and (a) surface ζ potential (ζ) measured at pH = 6, (b) on the pure PMMA composite material (black columns) and PMMA composite with two different concentrations of added CuO NPs (red columns) and ZnO NPs (blue columns), and (c) ζ potential of pure CuO and ZnO NPs measured at pH = 6. Significance was determined using a one-way ANOVA Tukey’s test with **** *p* < 0.0001. PMMA—poly(methyl methacrylate).

The water contact angle measurements on the surface of the PMMA (Figure 2a) demonstrated that the surfaces without NPs exhibited hydrophilic characteristics, with an average contact angle of (71.1 ± 2.4)°. After the addition of the NPs to the PMMA resin, no statistically significant change occurred regarding the hydrophobicity of the samples. Overall, the samples can be considered to be hydrophilic.

Furthermore, the surface ζ potentials of the samples (Figure 2b) were negative. The PMMA surface exhibited a surface potential of (−61.5 ± 1.1) mV. After the addition of the CuO and ZnO NPs, the ζ potential increased. One-way ANOVA analysis of significance demonstrated that the increase was statistically significant (*p* < 0.0001). The PMMA samples with 5 wt % of CuO (*p* < 0.0001) and ZnO (*p* < 0.0001) NPs exhibited higher surface potential than the samples with 2 wt % of NPs.

The color properties of the PMMA samples were measured, and the color change due to the addition of ZnO and CuO NPs was investigated using CIE colorimetry. The CIELAB system space is three dimensional, and it expresses color as three values: L* indicates lightness on a scale from black (0) to white (100), a* determines the ratio of green (negative) to red (positive), and b* determines the blue (negative) to yellow (positive) ratio. CIE L*, a*, and b* parameters of PMMA resin with and without CuO and ZnO NPs are presented in Table 1. Pure PMMA has the following CIE parameters: L* 44.31 ± 2.13; a*, 9.21 ± 0.05; b*, 2.85 ± 0.18. The addition of CuO NPs (having black color) significantly lowers the L*value, i.e., makes the material darker. Moreover, adding CuO NPs lowers a* value, causing the color to be less red. No change was observed for the b* parameter, which was only slightly increased since the sample became slightly more yellow. Regarding the quantity of added CuO NPs, there is no difference in L*, while 5 wt % of CuO NPs lowers the a* value (from 2.05 to 1.07) and also lowers the b* value (from 3.37 to 0.27).

**Table 1:** CIE L*, a*, b* Color Parameters of PMMA Doped with CuO and ZnO Nanoparticles

sample	L^*^	a^*^	b^*^
PMMA	44.31 ± 2.13	9.21 ± 0.05	2.85 ± 0.18
2 wt % CuO	26.78 ± 0.56	2.05 ± 0.08	3.37 ± 0.22
5 wt % CuO	28.26 ± 0.29	1.07 ± 0.40	0.27 ± 0.29
2 wt % ZnO	73.15 ± 0.11	13.86 ± 0.20	4.75 ± 0.47
5 wt % ZnO	49.25 ± 0.50	13.10 ± 0.28	3.30 ± 0.42

**Figure 3:**
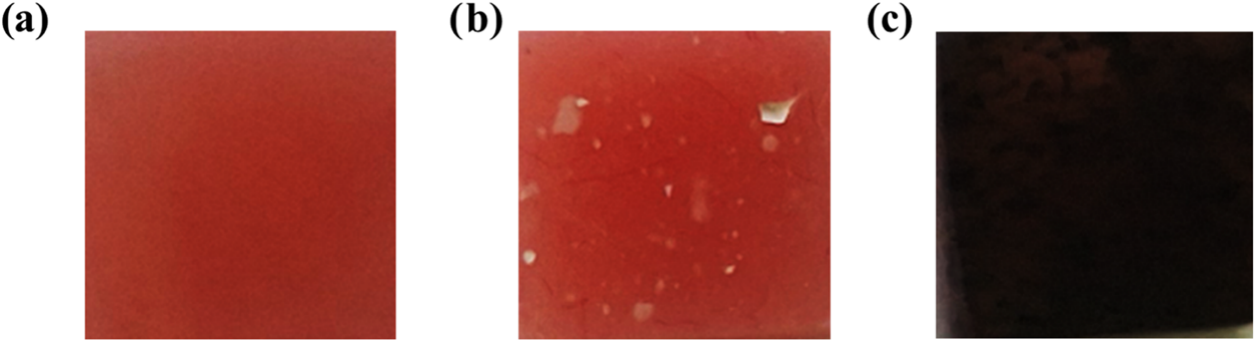
Photo of (a) blank PMMA, (b) PMMA with 5 wt % ZnO added, and (c) PMMA with 5 wt % CuO added.

In the PMMA samples with 5 wt % of ZnO NPs (having white color), a decrease in the L* value (from 73.15 to 49.25) and b* value (from 4.75 to 3.30) occurred. Color parameter a* does not differ between 2 and 5 wt % addition of ZnO NPs. Figure 3 shows how the materials are visually looking. The addition of 5 wt % ZnO did not change the color substantially. However, the addition of 5 wt % CuO into PMMA changed the color substantially to dark black.

**Figure 4:**
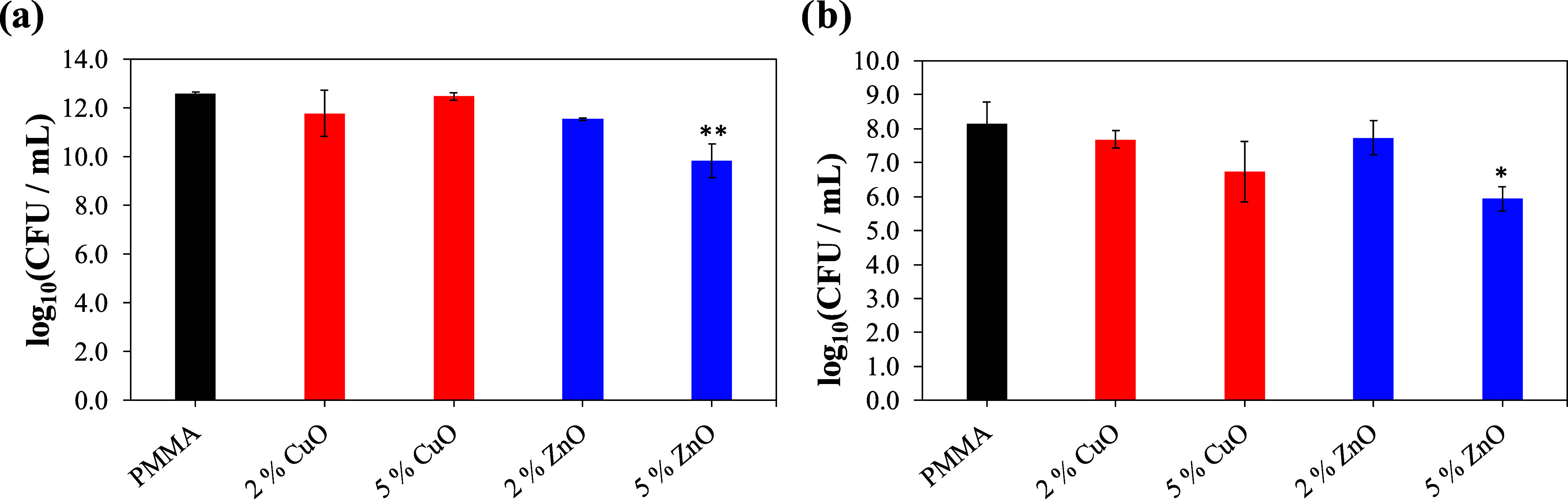
Number of attached viable cells (log(CFU mL^–1^)) on pure PMMA surfaces and PMMA surfaces doped with CuO and ZnO NPs in the case of (a) *Staphylococcus aureus* and (b) *Candida albicans*, represented as means with standard deviation bars. Significance was determined using a one-way ANOVA Tukey’s test of the log reduction data points with * *p* < 0.0146 and ** *p* ≤ 0.0063. PMMA—poly(methyl methacrylate). The experiments were conducted in triplicate, *n* = 3.

The antimicrobial tests of the studied materials were conducted with bacteria *S. aureus* (Figure 4a) and yeast *C. albicans* (Figure 4b) as described in Materials and Methods (2.3) by the addition of 100 μL of a microbial suspension (10^5^ cells/mL) onto the surface of the material samples in 24-well plates. After the 24 h incubation at 37 °C in the dark, the number of attached viable cells was determined. In the case of *S. aureus* the number of attached viable cells on doped PMMA materials compared with nondoped PMMA decreased in the case of 5 wt % ZnO NPs but not in the case of 2 wt % ZnO NPs as well as both concentrations of CuO (Figure 4; Table 2). Indeed, the one-way analysis of variance (ANOVA) demonstrated that the number of viable *S. aureus* cells on the pure PMMA samples did not significantly differ from the samples with 2 wt % CuO (*p* = 0.7069), 5 wt % CuO (*p* = 0.9997), and 2 wt % ZnO (*p* = 0.5172). However, as shown in Table 2, the PMMA doped with 5 wt % ZnO caused a decrease in the number of attached viable *S. aureus* cells by 87.2 ± 18.9% (*p* = 0.0109).

**Table 2:** Percentage Reduction of the Number of Attached Viable Cells of Bacteria *Staphylococcus aureus* and Yeast *Candida albicans* on the PMMA Surface Doped with CuO and ZnO Nanoparticles Compared with Undoped PMMA Samples^a^4

PMMA doped with	*Staphylococcus aureus*, %	*Candida albicans*, %
2 wt % CuO	32.3 ± 22.1	77.5 ± 15.9
5 wt % CuO	26.1 ± 15.6	82.3 ± 23.8
2 wt % ZnO	71.9 ± 16.7	63.4 ± 15.0
5 wt % ZnO	87.2 ± 18.9	88.0 ± 13.4

a The experiments were conducted in triplicate, *n* = 3 and the mean percentage reduction with standard deviation is presented. See also Figure 4.

In the case of *C. albicans*, the decrease in the number of attached viable cells by more than 60% was observed in the case of all studied doped-PMMA samples, whereas the most efficient inhibition (88 ± 13.4)% was observed for 5 wt % ZnO-doped PMMA (Table 2, Figure 4b) (*p* = 0.0063). For other combinations, the inhibition was not statistically significant from the PMMA sample: 2 wt % CuO (p = 0.8736), 5 wt % CuO (*p* = 0.0813), and 2 wt % ZnO (*p* = 0.9064). Overall, the samples with 5 wt % of ZnO demonstrated the best antimicrobial performance.

**Figure 5:**
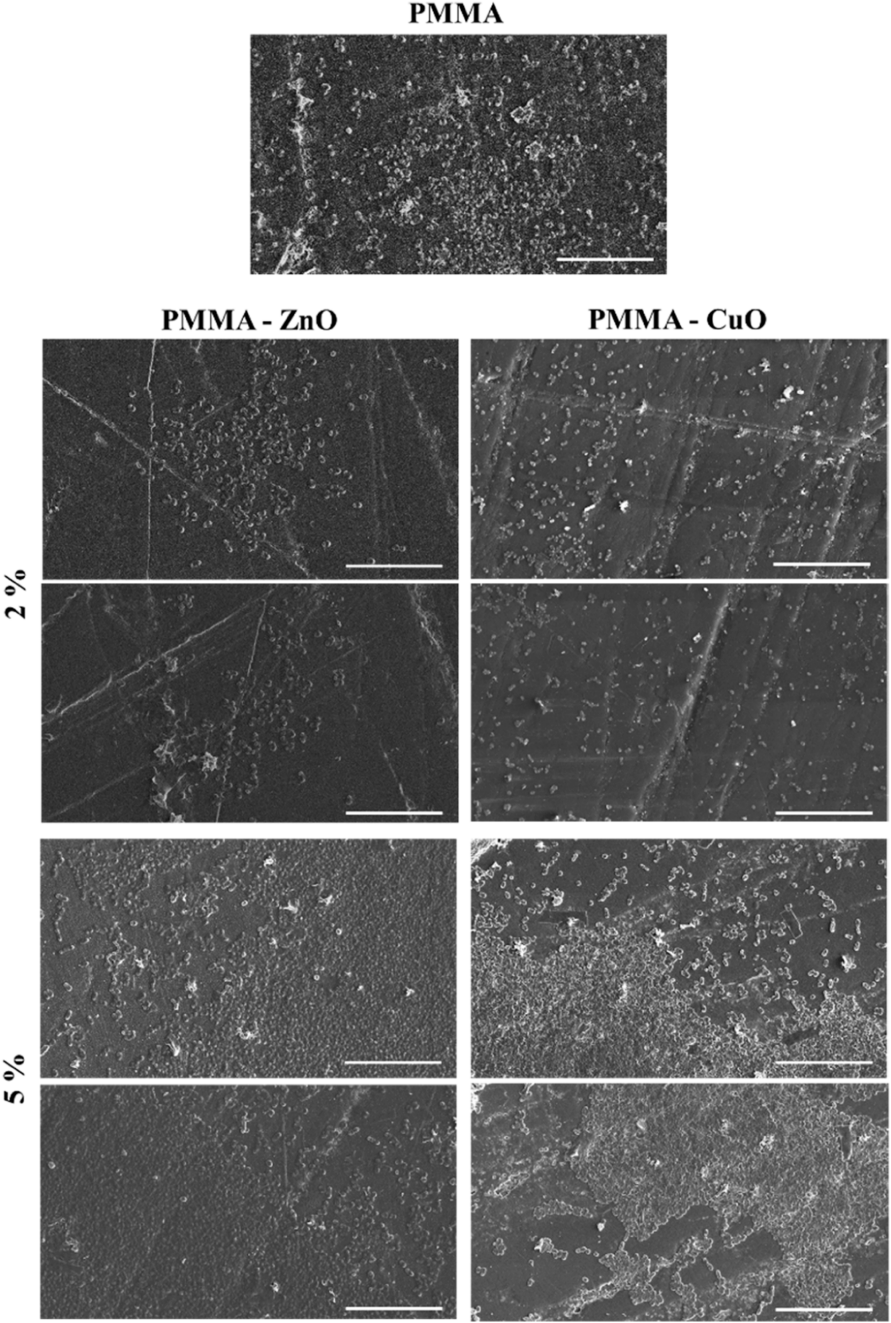
SEM micrographs of *Candida albicans* after 24 h of incubation time. The micrographs are made on pure PMMA surfaces and two representative images on PMMA surfaces doped with 2 and 5 wt % ZnO or CuO nanoparticles. Scale bar is 100 μm.

In addition, after the incubation of the PMMA surfaces with added *C. albicanssuspensions*, the surface of the samples was analyzed with SEM to visualize the adhered cells (Figure 5): the number of the adhered cells on PMMA surfaces doped with either 2 wt % ZnO or 2 wt % CuO slightly decreased but a slight increase of adhered cells was observed on the surface of the PMMA samples with 5 wt % CuO and ZnO.

## 4 Discussion

The aim of this study was to evaluate whether the ZnO- or CuO-doped PMMA materials could be applied as a base material for dentures. One of the most intensive focuses of scientists is related to the use of metal-based nanoparticles in biomedical sciences and engineering. Due to their antimicrobial activity against Gram-positive and Gram-negative bacteria, these particles have been proposed as an alternative to conventional drugs, especially to overcome microbial resistance. Nanoparticles are known to use multiple, atypical mechanisms of action, unlike conventional drugs, which prevent microorganisms from developing resistance to their action.^20^ For that, the PMMA materials doped with ZnO or CuO NPs were synthesized and studied for (i) color properties, surface ζ potential, and contact angle, and (ii) antimicrobial and antiadhesive properties toward Gram-positive bacteria *S. aureus* and yeast *C. albicans*.

Regarding quantitative color measurements of dentistry restorative material, CIE (the International Commission on Illumination) L*, a*, b* color parameters proved a reliable method for distinguishing differences between the gingiva and restorative material.^17,19,46^ The addition of CuO NPs substantially changed the color of PMMA by lowering the L*value and thus making the material darker, but no changes were observed between 2 wt % CuO and 5 wt % Cuo. Moreover, the addition of 2 wt % of CuO NPs also lowered a* value, turning the color toward less red tint. Again, no additional lowering of a* value occurred at 5 wt % Cuo. The addition of 2 wt % ZnO NPs increased L*, while 5 wt % ZnO NPs resulted in a similar L* value as pure PMMA. The addition of ZnO NPs slightly increased a* and b* parameters making the PMMA doped with CuO and ZnO nanoparticles slightly more reddish and more intensive yellow. According to Ho et al. human gingiva has on average the following CIE color coordinates L* 52.9, a* 23.3, and b* 14.9,^47^ the addition of CuO NPs changes the color beyond the acceptable range to match human gingiva color. The addition of NPs along with the change of color can also affect the PMMA mechanical properties. The tensile stress performance of PMMA showed that the maximum tensile stress decreases with an increasing amount of added metal NPs,^17^ which is expected to occur with the addition of ZnO and CuO NPs as well.

The addition of ZnO or CuO to PMMA changed the ζ potential to less negative. Namely, the pure PMMA surface exhibited a surface potential of (−61.5 ± 1.1) mV. After the addition of the negatively charged CuO (−53 mV) and ZnO (−47 mV) NPs, the ζ potential increased. Similar observations were found by other researchers for materials such as textiles with more negative charges (−300 mV) coated with CuO NPs.^38^

The water contact angle measurements revealed that the PMMA surface is hydrophilic. After the addition of the CuO and ZnO NPs to the PMMA resin, no statistically significant change occurred regarding the hydrophilicity of the PMMA samples. Similar findings were obtained with Au and TiO_2_ NPs used as dopants.^17,19^

The adhesion of microbial cells to the material’s surface depends on the overall surface charge and hydrophobicity. As mentioned above, the PMMA surfaces with and without CuO and ZnO NPs exhibited hydrophilic characteristics with no expressed differences between samples (Figure 2a). It is well-known that microbial cells tend to adhere to hydrophilic surfaces^48−51^ but as in our case all the studied surfaces were hydrophilic (Figure 1a) also the surface charge of the surfaces must be taken into account. The PMMA surface exhibited a negative surface charge, which increased with the addition of the CuO and ZnO NPs (Figure 2b). *S. aureus* cells have an overall negative net surface charge since the cell’s lipid bilayers contain teichoic acid^52^ and phosphate heads.^53^ Analogously, *C. albicans* and most of the fungal cells also have a negative surface charge.^54,55^ Due to the difference in the overall surface charge, microbial cells adhered more significantly to PMMA surfaces with 5 wt % CuO and 5 wt % ZnO having more positive surface charge (Figure 5). This is in agreement with the previous research that has demonstrated that negatively charged microbial cells adhere less to negatively charged surfaces due to repulsive electrostatic forces.^56,57^ The repulsion between negatively charged bacteria and negatively charged material surfaces as a consequence leads to lower bacterial adhesion extent.^49^ It can be assumed that due to the higher adhesion of the microbial cells to the PMMA surfaces with 5 wt % of NPs the antibacterial effect of CuO and ZnO NPs was more pronounced (Figure 4, Table 2). Overall, ZnO NPs caused a more significant antibacterial effect than did CuO NPs. Previous studies^24,58^ also showed that ZnO NPs caused higher antibacterial effects than CuO NPs. Also, Ginjupalli et al. used 5 wt % CuO and ZnO NPs for the production of alginate dental impression materials and demonstrated higher antibacterial activity with ZnO NPs.^59^ In addition to individual action, nanoforms of ZnO and CuO show a synergistic antimicrobial mechanism in combination with graphene.^60,61^ Khlifi et al. synthesized ZnO NPs and added them to conventional PMMA for the manufacture of orthodontic appliances, in concentrations of 1, 2, and 3 wt %. They concluded that the newly formed PMMA/ZnO NP composite shows high antimicrobial activity against *E. coli* and *S. aureus*. The results of the study suggest that PMMA modified with the addition of ZnO NPs shows promising potential in the production of PMMA/ZnO with high antimicrobial performance for orthodontic appliances.^62^

A study by Giti et al. studied the effect of PMMA modification with the addition of 2.5 and 7.5 wt % copper oxide (CuO) and titanium dioxide (TiO_2_) nanoparticles after thermocycling on antimicrobial activity against standard strains of *C. albicans*, *Candida dubliniensis* and oral species *Streptococcus mutans*, *Streptococcus sobrius*, *Streptococcus salivarius*, and *Streptococcus sanguis*. The results showed that both concentrations of CuO and TiO_2_ have significant antimicrobial activity against *Streptococcus salivarius*, *Streptococcus sanguis*, and *Candida dubliniensis* compared to the control group.^32^ None of the tested concentrations of CuO nanoparticles showed antimicrobial activity against *C. albicans*, which is consistent with the results of our study.

By generating reactive oxygen species ROS and inhibiting bacteria in contact with the cell wall, ZnO NPs exhibit antibacterial properties in inhibiting biofilm formation. Padmavathy et al. investigated the antibacterial effect of ZnO NPs on various microorganisms, and the mechanism of action was described for Gram-negative *E. coli* as a model organism by analyzing the growth, permeability, and morphology of bacterial cells.^63^ The study showed that ZnO NPs damage the structure of the bacterial cell membrane and reduce the activity of some membrane enzymes by producing ROS, which ultimately causes the death of *E. coli* bacteria. The authors conclude that the antibacterial mechanism of ZnO NPs occurs through multiple mechanisms such as increasing the permeability of the outer membrane, which leads to the leakage of cellular materials.^64^

Research has proven the difference in the antimicrobial activity of ZnO nanoparticles against Gram-positive and Gram-negative microorganisms, which originates from the different structures of the cell wall. The liposaccharide structure of Gram-negative bacteria provides resistance to the binding and passage of ZnO ions.^65^ The mentioned fact can explain the positive antimicrobial effect of ZnO nanoparticles on Gram-positive *S. aureus*. It is believed that, due to their positive charge, Zn^2+^ ions can more easily penetrate through the bacterial wall and interfere with the basic metabolic processes inside the microorganism cell, interacting with proteins, lipids, and nucleic acids. In an environment with low pH, nanoparticles show greater antimicrobial activity due to enhanced dissolution and release of a larger amount of Zn^2+^ ions.^66^ The mentioned information could be of importance, presenting an additional mechanism of action of ZnO nanoparticles, if it is taken into account that the acidic environment favors microbial activity and that it often develops in the oral cavity. Numerous studies have also proven the antimicrobial activity of CuO nanoforms.^34−36^ The mechanism of the antibacterial activity of CuO NPs has not yet been fully explained, but it is believed to be based on the adhesion of nanoions to the cell wall of the microorganism caused by electrostatic forces. The mechanism of action is multiple and includes the development of ROS oxidative stress, membrane disruption, destruction of internal contents, and its leakage outside the cell.^67,68^

It can be assumed that due to the higher adhesion of the microbial cells to the PMMA surfaces with 5 wt % of NPs, the antibacterial effect of CuO and ZnO NPs was more pronounced (Figure 4, Table 2). The current level of antimicrobial activity may already be beneficial in reducing microbial colonization and could serve as a supplementary measure to improve hygiene and reduce infection risks for denture wearers. Overall, ZnO NPs caused a more significant antibacterial effect than the CuO NPs. All-in-all, our study showed that 5 wt % ZnO added to PMMA yields promising denture material that is esthetically acceptable and has antimicrobial properties toward *S. aureus* and *C. albicans*. The antimicrobial properties of produced PMMA could be further enhanced by optimizing the concentration of ZnO and CuO nanoparticles, as higher amounts may increase efficacy. However, this must be balanced with preserving the material mechanical properties, aesthetic quality, and biocompatibility. Additional antimicrobial agents, such as silver nanoparticles or natural compounds, could work synergistically with ZnO and CuO, while surface modifications or coatings may improve nanoparticle retention and microbial interaction. Furthermore, advanced processing techniques like plasma treatment or UV activation could activate the surface nanoparticles, further boosting their antibacterial activity. While this study provides valuable insights into the antimicrobial properties of PMMA composites doped with ZnO and CuO nanoparticles, several limitations should be acknowledged. First, the particle size of the nanoparticles was determined using SEM and DLS, but TEM analysis was not conducted, which could have provided additional information on particle morphology and size distribution at a finer resolution. Second, while the antimicrobial efficacy of the composites was demonstrated, the specific mechanisms of action, such as reactive oxygen species (ROS) generation or lipid peroxidation, were not explored in detail, as these mechanisms have been well-documented in previous studies. Future research could focus on these aspects to provide a more comprehensive understanding of the material’s antimicrobial behavior. Further insights into the practical performance of the materials in real-world scenarios of the produced materials and future research should focus on the durability and stability of the antimicrobial properties of the PMMA composites under simulated conditions, such as exposure to saliva, food, and varying temperatures.

## 5 Conclusions

In this research, the antimicrobial effect of PMMA incorporated with different amounts (2 and 5 wt %) of zinc oxide and copper oxide was determined on Gram-positive bacteria *Staphylococcus aureus* and yeast *Candida albicans*, pathogenic microbes often found on dentures. The key findings are summarized below.

- The addition of CuO NPs substantially changed the color of PMMA by making the material darker and not acceptable for dentures. On the other hand, ZnO NPs did not change the color of PMMA significantly.
- The addition of ZnO or CuO caused an increase in the PMMA ζ potential.
- 2 and 5 wt % of CuO and 2 wt % ZnO NPs did not cause statistically significant antimicrobial properties toward *S. aureus* and *C. albicans*.
- 5 wt % ZnO added to PMMA yields a promising denture material that is esthetically acceptable and shows antimicrobial properties toward both, *S. aureus* and *C. albicans*.
- The antimicrobial effect of ZnO is much more pronounced than CuO-doped PMMA toward both, *S. aureus* and *C. albicans*.
